# Supplementary material for: Target Enrichment Metagenomics Reveals Human Pegivirus-1 in Pediatric Hematopoietic Stem Cell Transplantation Recipients
Source: Viruses. 2022 Apr 12;14(4):796. doi: 10.3390/v14040796 (PMC9025367; doi:10.3390/v14040796)
Supplement: Supplementary file 1 [file viruses-14-00796-s001.zip › viruses-1651788-supplementary.pdf]

Supplementary Tables

Table S1: qRT-PCR results and the Virus Identification Pipeline analysis output for the positive internal control, Newcastle Disease Virus (NDV).

Sample number	NDV particles (per 1ml blood)	% NDV coverage	Read hits	Average depth coverage	qRT-PCR
001-D0	$1{\times}10^4$	100	139,882	3,910.70	Positive
001-D3	$1{\times}10^4$	100	1,737,559	48,498.28	Positive
002-D0	$1{\times}10^4$	100	362,876	11,079.96	Positive
002-D3	$1{\times}10^4$	100	2,302,948	68,348.46	Positive
003-D0	$1{\times}10^4$	100	741,509	21,908.69	Positive
003-D3	$1{\times}10^4$	100	5,415	133.25	Positive
004-D0	$1{\times}10^4$	100	2,232,001	69759.43	Positive
004-D3	$1{\times}10^4$	100	21,688	661.89	Positive
005-D0	$1{\times}10^4$	100	268,477	8,058.97	Positive
006-D0	$1{\times}10^4$	99.28	6,762	191.35	Positive
006-D3	$1{\times}10^4$	100	1,057,996	32,375.66	Positive
007-D0	$1{\times}10^4$	100	69,031	1,839	Positive
007-D3	$1{\times}10^4$	100	841,928	21,960	Positive
008-D0	$1{\times}10^3$	100	76,091	2,180	Positive
008-D3	$1{\times}10^3$	99.59	7,035	159.86	Positive
009-D0	$1{\times}10^3$	100	12,533	304.65	Positive
009-D3	$1{\times}10^3$	99.95	27,649	697.15	Positive
011-D0	$1{\times}10^4$	100	603,461	17,672	Positive
011-D3	$1{\times}10^4$	100	758,192	23,067	Positive
012-D0	$1{\times}10^4$	100	3,165,402	96,396	Positive
012-D3	$1{\times}10^4$	100	574,423	15,378	Positive
013-D0	$1{\times}10^4$	100	138,532	4,116	Positive
013-D3	$1{\times}10^4$	100	102,209	3,057	Positive
015-DA	$1{\times}10^4$	100	85,325	2,117	Positive
015-D0	$1{\times}10^4$	100	843,550	24,990	Positive
015-D3	$1{\times}10^4$	100	30,992	931	Positive

Table S2: Sequencing read distribution as determined by Virus Identification Pipeline [1]

Sample code	Total number of reads (million)	Viral reads %	Host reads %	Other reads %	Bacterial reads %	Low quality reads %
001-D0	1.83	21.4	18.4	54.1	0.0	6.1
001-D3	3.76	48.4	40.3	7.9	0.0	3.4
002-DA	2.66	91.8	1.7	0.2	0.0	6.3
002-D0	2.11	28.5	26.6	39.4	0.0	5.5
002-D3	4.02	59.6	30.8	6.8	0.0	2.9
003-D0	2.46	32.0	59.1	5.2	0.0	3.8
003-D3	1.59	10.6	48.6	34.7	0.0	6.0
004-D0	3.91	58.8	33.7	5.1	0.0	2.4
004-D3	0.73	5.5	85.5	3.6	0.0	5.4
005-D0	1.90	16.2	74.4	4.7	0.0	4.7
006-D0	0.60	14.2	66.0	14.3	0.0	5.5
006-D3	2.63	42.8	43.4	9.2	0.0	4.6
007-D0	1.97	13.7	43.8	36.7	0.0	5.9
007-D3	1.05	82.1	6.0	9.8	0.0	2.1
008-D0	2.06	6.8	79.3	6.4	0.0	7.6
008-D3	2.19	2.8	87.1	4.5	0.0	5.6
009-D0	1.97	3.2	87.2	4.5	0.0	5.0
009-D3	1.27	2.5	88.7	3.7	0.0	5.1
011-DA	5.36	69.8	15.8	12.8	0.0	1.6
011-D0	1.86	36.8	53.3	6.1	0.0	3.8
011-D3	2.20	38.9	52.7	4.6	0.0	3.8
012-D0	4.61	72.1	12.8	12.7	0.0	2.5
012-D3	0.66	87.8	3.0	7.4	0.0	1.8
013-D0	1.49	12.7	74.6	7.9	0.0	4.8
013-D3	1.70	19.4	30.9	43.7	0.0	6.0
015-DA	1.04	15.5	76.5	4.5	0.0	3.5
015-D0	3.4	59.4	2.1	36.2	0.0	2.3
015-D3	0.19	43.9	21.2	32.1	0.0	2.8

Table S3: Polymorphic sites in the recovered human pegivirus-1 genome sequences

Sample	Position with respect to the reference genome NC_001710.1	Variant (%)			
		A	T	C	G
002_D0	363	0.00	40.48	59.52	0.00
002_D0	796	77.84	0.00	0.00	22.16
002_D0	896	69.42	0.00	0.00	30.58
002_D0	922	0.00	75.65	24.35	0.00
002_D0	962	0.00	22.04	77.96	0.00
002_D0	1075	0.00	69.89	0.00	30.11
002_D0	1378	0.00	71.60	28.40	0.00
002_D0	1471	0.00	57.05	42.95	0.00
002_D0	1503	69.05	0.00	0.00	30.95
002_D0	1510	0.00	73.35	26.65	0.00
002_D0	1525	71.88	0.00	0.00	28.13
002_D0	1530	0.00	29.68	70.32	0.00
002_D0	1561	70.60	0.00	0.00	29.40
002_D0	1882	0.00	33.10	66.90	0.00
002_D0	2482	28.00	0.00	0.00	72.00
002_D0	2488	0.00	27.84	72.16	0.00
002_D0	2521	0.00	68.68	31.32	0.00
002_D0	2527	0.00	25.79	74.21	0.00
002_D0	2704	0.00	72.84	27.16	0.00
002_D0	2755	0.00	78.66	21.34	0.00
002_D0	2803	82.18	0.00	0.00	17.82
002_D0	2809	0.00	65.93	34.07	0.00
002_D0	2831	0.00	33.33	66.67	0.00
002_D0	3001	0.00	78.02	21.98	0.00
002_D0	3002	0.00	79.67	20.33	0.00
002_D0	3025	0.00	21.93	78.07	0.00
002_D0	3049	0.00	78.71	21.29	0.00
002_D0	3121	16.04	72.64	0.00	11.32
002_D0	3172	0.00	67.61	32.39	0.00
002_D0	3238	0.00	29.21	70.79	0.00
002_D0	3320	0.00	71.65	28.35	0.00
002_D0	3418	37.11	0.00	0.00	62.89
002_D0	3451	0.00	37.69	62.31	0.00
002_D0	3577	0.00	76.68	23.32	0.00
002_D0	3742	0.00	28.65	71.35	0.00
002_D0	3772	0.00	77.59	22.41	0.00
002_D0	3928	61.36	0.00	0.00	38.64
002_D0	4000	0.00	76.58	23.42	0.00
002_D0	4051	0.00	26.72	73.28	0.00
002_D0	4228	0.00	67.18	32.82	0.00
002_D0	4525	34.69	0.00	0.00	65.31
002_D0	4660	0.00	65.79	34.21	0.00
002_D0	4693	0.00	28.03	71.97	0.00
002_D0	4724	64.77	0.00	0.00	35.23
002_D0	4837	28.47	0.00	0.00	71.53
002_D0	5377	0.00	36.55	63.45	0.00
002_D0	5461	24.83	0.00	0.00	75.17
002_D0	5479	0.00	62.17	37.83	0.00
002_D0	5551	72.84	0.00	0.00	27.16
002_D0	5735	0.00	39.13	60.87	0.00
002_D0	5767	0.00	67.47	32.53	0.00
002_D0	5833	0.00	38.04	61.96	0.00
002_D0	5902	55.07	0.00	0.00	44.93
002_D0	5920	44.44	0.00	0.00	55.56
002_D0	6082	72.95	0.00	0.00	27.05
002_D0	6145	0.00	30.36	69.64	0.00
002_D0	6199	70.24	0.00	0.00	29.76
002_D0	6238	79.84	0.00	0.00	20.16
002_D0	6490	0.00	67.86	32.14	0.00
002_D0	6619	0.00	26.71	73.29	0.00

002_D0	6673	74.87	0.00	0.00	25.13
002_D0	6727	0.00	19.70	80.30	0.00
002_D0	6844	0.00	39.28	60.72	0.00
002_D0	6874	78.91	0.00	0.00	21.09
002_D0	7003	0.00	77.13	22.87	0.00
002_D0	7021	61.02	0.00	0.00	38.98
002_D0	7120	67.84	32.16	0.00	0.00
002_D0	7210	0.00	28.93	71.07	0.00
002_D0	7222	0.00	28.79	71.21	0.00
002_D0	7266	56.45	0.00	0.00	43.55
002_D0	7441	79.08	20.92	0.00	0.00
002_D0	7486	67.77	0.00	0.00	32.23
002_D0	7645	63.80	0.00	0.00	36.20
002_D0	7735	0.00	51.97	48.03	0.00
002_D0	7957	31.60	0.00	0.00	68.40
002_D0	8005	26.78	0.00	0.00	73.22
002_D0	8161	0.00	75.98	24.02	0.00
002_D0	8263	0.00	65.03	34.97	0.00
002_D0	8359	0.00	32.41	67.59	0.00
002_D0	8411	69.23	0.00	0.00	30.77
002_D0	8413	69.13	0.00	0.00	30.87
002_D0	8461	0.00	70.22	29.78	0.00
002_D0	8467	0.00	68.06	31.94	0.00
002_D0	8470	30.83	0.00	0.00	69.17
002_D0	8605	0.00	30.76	69.24	0.00
002_D3	307	0.00	76.74	23.26	0.00
002_D3	363	0.00	43.90	56.10	0.00
002_D3	588	0.00	71.43	28.57	0.00
002_D3	896	69.85	0.00	0.00	30.15
002_D3	922	0.00	73.68	26.32	0.00
002_D3	962	0.00	24.45	75.55	0.00
002_D3	1075	0.00	70.34	0.00	29.66
002_D3	1378	0.00	76.42	23.58	0.00
002_D3	1471	0.00	54.68	45.32	0.00
002_D3	1503	65.14	0.00	0.00	34.86
002_D3	1510	0.00	74.14	25.86	0.00
002_D3	1525	71.17	0.00	0.00	28.83
002_D3	1530	0.00	30.61	69.39	0.00
002_D3	1561	64.51	0.00	0.00	35.49
002_D3	1882	0.00	29.81	70.19	0.00
002_D3	2482	27.39	0.00	0.00	72.61
002_D3	2488	0.00	22.22	77.78	0.00
002_D3	2521	0.00	68.57	31.43	0.00
002_D3	2704	0.00	77.08	22.92	0.00
002_D3	2755	0.00	67.92	32.08	0.00
002_D3	2809	0.00	66.40	33.60	0.00
002_D3	2831	0.00	31.09	68.91	0.00
002_D3	3001	0.00	66.67	33.33	0.00
002_D3	3002	0.00	67.38	32.62	0.00
002_D3	3025	0.00	30.67	69.33	0.00
002_D3	3049	0.00	65.85	34.15	0.00
002_D3	3121	16.33	83.67	0.00	0.00
002_D3	3172	0.00	57.83	42.17	0.00
002_D3	3238	0.00	29.55	70.45	0.00
002_D3	3320	0.00	71.68	28.32	0.00
002_D3	3418	27.85	0.00	0.00	72.15
002_D3	3451	0.00	26.69	73.31	0.00
002_D3	3577	0.00	74.21	25.79	0.00
002_D3	3928	69.31	0.00	0.00	30.69
002_D3	4000	0.00	54.55	45.45	0.00
002_D3	4051	0.00	35.06	64.94	0.00
002_D3	4228	0.00	73.19	26.81	0.00
002_D3	4525	35.79	0.00	0.00	64.21
002_D3	4660	0.00	62.28	37.72	0.00
002_D3	4693	0.00	33.14	66.86	0.00
002_D3	4724	67.18	0.00	0.00	32.82

002_D3	4837	25.23	0.00	0.00	74.77
002_D3	5377	0.00	34.48	65.52	0.00
002_D3	5461	24.08	0.00	0.00	75.92
002_D3	5479	0.00	59.60	40.40	0.00
002_D3	5735	0.00	37.50	62.50	0.00
002_D3	5767	0.00	76.12	23.88	0.00
002_D3	5833	0.00	28.72	71.28	0.00
002_D3	5902	73.78	0.00	0.00	26.22
002_D3	5920	58.86	0.00	0.00	41.14
002_D3	6082	75.12	0.00	0.00	24.88
002_D3	6145	0.00	30.10	69.90	0.00
002_D3	6199	70.62	0.00	0.00	29.38
002_D3	6238	66.49	0.00	0.00	33.51
002_D3	6490	0.00	72.37	27.63	0.00
002_D3	6619	0.00	36.86	63.14	0.00
002_D3	6673	68.60	0.00	0.00	31.40
002_D3	6727	0.00	20.34	79.66	0.00
002_D3	6844	0.00	39.87	60.13	0.00
002_D3	6874	79.00	0.00	0.00	21.00
002_D3	7003	0.00	79.51	20.49	0.00
002_D3	7021	59.87	0.00	0.00	40.13
002_D3	7120	67.09	32.91	0.00	0.00
002_D3	7210	0.00	26.11	73.89	0.00
002_D3	7222	0.00	30.51	69.49	0.00
002_D3	7266	55.05	0.00	0.00	44.95
002_D3	7303	22.14	0.00	0.00	77.86
002_D3	7441	79.09	20.91	0.00	0.00
002_D3	7486	68.35	0.00	0.00	31.65
002_D3	7645	67.70	0.00	0.00	32.30
002_D3	7735	0.00	51.02	48.98	0.00
002_D3	7957	25.60	0.00	0.00	74.40
002_D3	8005	21.43	0.00	0.00	78.57
002_D3	8161	0.00	77.23	22.77	0.00
002_D3	8263	0.00	65.57	34.43	0.00
002_D3	8359	0.00	32.75	67.25	0.00
002_D3	8411	70.69	0.00	0.00	29.31
002_D3	8413	69.29	0.00	0.00	30.71
002_D3	8461	0.00	75.11	24.89	0.00
002_D3	8467	0.00	73.86	26.14	0.00
002_D3	8470	25.11	0.00	0.00	74.89
002_D3	8605	0.00	27.84	72.16	0.00
002_D3	8842	0.00	34.62	65.38	0.00
002_D3	8863	0.00	34.78	65.22	0.00
002_DA	588	0.00	74.29	25.71	0.00
002_DA	896	65.64	0.00	0.00	34.36
002_DA	922	0.00	67.25	32.75	0.00
002_DA	962	0.00	24.36	75.64	0.00
002_DA	1075	0.00	69.65	0.00	30.35
002_DA	1378	0.00	71.57	28.43	0.00
002_DA	1471	0.00	58.41	41.59	0.00
002_DA	1503	62.63	0.00	0.00	37.37
002_DA	1510	0.00	71.02	28.98	0.00
002_DA	1525	71.46	0.00	0.00	28.54
002_DA	1530	0.00	31.30	68.70	0.00
002_DA	1561	65.95	0.00	0.00	34.05
002_DA	1882	0.00	34.40	65.60	0.00
002_DA	2482	21.24	0.00	0.00	78.76
002_DA	2521	0.00	65.90	34.10	0.00
002_DA	2527	0.00	20.34	79.66	0.00
002_DA	2704	0.00	75.89	24.11	0.00
002_DA	2755	0.00	70.19	29.81	0.00
002_DA	2809	0.00	64.29	35.71	0.00
002_DA	2831	0.00	31.11	68.89	0.00
002_DA	3001	0.00	77.12	22.88	0.00
002_DA	3002	0.00	75.66	24.34	0.00
002_DA	3025	0.00	28.83	71.17	0.00

002_DA	3049	0.00	67.66	32.34	0.00
002_DA	3121	17.86	66.96	0.00	15.18
002_DA	3172	0.00	72.34	27.66	0.00
002_DA	3238	0.00	28.92	71.08	0.00
002_DA	3320	0.00	70.39	29.61	0.00
002_DA	3418	35.24	0.00	0.00	64.76
002_DA	3451	0.00	30.65	69.35	0.00
002_DA	3577	0.00	79.70	20.30	0.00
002_DA	3742	0.00	28.57	71.43	0.00
002_DA	3772	0.00	76.64	23.36	0.00
002_DA	3928	60.27	0.00	0.00	39.73
002_DA	4000	0.00	69.30	30.70	0.00
002_DA	4051	0.00	23.66	76.34	0.00
002_DA	4228	0.00	66.31	33.69	0.00
002_DA	4525	34.73	0.00	0.00	65.27
002_DA	4660	0.00	66.15	33.85	0.00
002_DA	4693	0.00	28.65	71.35	0.00
002_DA	4724	63.93	0.00	0.00	36.07
002_DA	4837	27.31	0.00	0.00	72.69
002_DA	5377	0.00	34.39	65.61	0.00
002_DA	5461	25.28	0.00	0.00	74.72
002_DA	5479	0.00	64.17	35.83	0.00
002_DA	5551	74.61	0.00	0.00	25.39
002_DA	5602	23.13	0.00	0.00	76.88
002_DA	5735	0.00	58.82	41.18	0.00
002_DA	5767	0.00	65.28	34.72	0.00
002_DA	5833	0.00	29.46	70.54	0.00
002_DA	5902	62.22	0.00	0.00	37.78
002_DA	5920	57.09	0.00	0.00	42.91
002_DA	6082	76.07	0.00	0.00	23.93
002_DA	6145	0.00	30.13	69.87	0.00
002_DA	6199	73.47	0.00	0.00	26.53
002_DA	6490	0.00	75.96	24.04	0.00
002_DA	6619	0.00	32.68	67.32	0.00
002_DA	6673	69.36	0.00	0.00	30.64
002_DA	6727	0.00	20.33	79.67	0.00
002_DA	6844	0.00	40.40	59.60	0.00
002_DA	6874	78.04	0.00	0.00	21.96
002_DA	7003	0.00	75.10	24.90	0.00
002_DA	7021	59.40	0.00	0.00	40.60
002_DA	7120	72.30	27.70	0.00	0.00
002_DA	7210	0.00	25.96	74.04	0.00
002_DA	7222	0.00	27.91	72.09	0.00
002_DA	7266	59.88	0.00	0.00	40.12
002_DA	7441	80.80	19.20	0.00	0.00
002_DA	7486	70.18	0.00	0.00	29.82
002_DA	7645	68.47	0.00	0.00	31.53
002_DA	7735	0.00	50.71	49.29	0.00
002_DA	7957	33.82	0.00	0.00	66.18
002_DA	8005	26.39	0.00	0.00	73.61
002_DA	8161	0.00	77.11	22.89	0.00
002_DA	8263	0.00	67.11	32.89	0.00
002_DA	8359	0.00	32.67	67.33	0.00
002_DA	8411	70.44	0.00	0.00	29.56
002_DA	8413	69.58	0.00	0.00	30.42
002_DA	8461	0.00	73.47	26.53	0.00
002_DA	8467	0.00	70.23	29.77	0.00
002_DA	8470	29.02	0.00	0.00	70.98
002_DA	8605	0.00	29.40	70.60	0.00
011_D0	546	0.00	71.81	28.19	0.00
011_D0	628	0.00	78.61	21.39	0.00
011_D0	634	76.87	0.00	0.00	23.13
011_D0	2002	0.00	34.40	65.60	0.00
011_D0	2131	0.00	25.09	74.91	0.00
011_D0	2380	0.00	38.86	61.14	0.00
011_D0	2389	0.00	60.75	39.25	0.00

011_D0	2500	31.71	0.00	0.00	68.29
011_D0	2711	0.00	27.21	72.79	0.00
011_D0	2807	56.56	0.00	0.00	43.44
011_D0	3007	0.00	73.71	26.29	0.00
011_D0	3480	77.67	22.33	0.00	0.00
011_D0	3778	55.33	0.00	0.00	44.67
011_D0	3895	0.00	20.89	79.11	0.00
011_D0	4024	56.68	0.00	0.00	43.32
011_D0	4219	0.00	31.03	68.97	0.00
011_D0	4228	0.00	56.03	0.00	43.97
011_D0	4396	0.00	19.82	80.18	0.00
011_D0	5275	0.00	46.76	53.24	0.00
011_D0	5587	0.00	33.53	66.47	0.00
011_D0	5701	20.88	0.00	0.00	79.12
011_D0	5842	0.00	73.06	26.94	0.00
011_D0	5929	0.00	29.33	70.67	0.00
011_D0	5942	0.00	77.09	22.91	0.00
011_D0	6061	0.00	74.86	25.14	0.00
011_D0	6124	0.00	21.95	78.05	0.00
011_D0	6259	0.00	45.48	54.52	0.00
011_D0	7513	25.70	0.00	0.00	74.30
011_D0	7546	78.91	0.00	0.00	21.09
011_D0	7648	0.00	34.55	65.45	0.00
011_D0	7816	0.00	76.15	23.85	0.00
011_D0	7915	0.00	79.25	20.75	0.00
011_D0	7978	68.18	0.00	0.00	31.82
011_D0	8014	0.00	50.54	49.46	0.00
011_D0	8046	73.63	0.00	0.00	26.37
011_D0	8059	31.34	0.00	0.00	68.66
011_D0	8071	79.45	0.00	0.00	20.55
011_D0	8080	0.00	77.77	22.23	0.00
011_D0	8095	0.00	79.63	20.37	0.00
011_D0	8101	0.00	74.08	25.92	0.00
011_D0	8317	0.00	77.65	22.35	0.00
011_D0	8419	0.00	75.82	24.18	0.00
011_D0	8692	0.00	70.25	29.75	0.00
011_D3	546	0.00	57.84	42.16	0.00
011_D3	634	79.71	0.00	0.00	20.29
011_D3	1165	25.48	0.00	0.00	74.52
011_D3	1256	21.76	0.00	0.00	78.24
011_D3	1449	79.49	0.00	20.51	0.00
011_D3	2002	0.00	35.57	64.43	0.00
011_D3	2131	0.00	20.50	79.50	0.00
011_D3	2380	0.00	42.83	57.17	0.00
011_D3	2389	0.00	54.49	45.51	0.00
011_D3	2417	0.00	21.88	78.12	0.00
011_D3	2500	43.88	0.00	0.00	56.12
011_D3	2807	53.27	0.00	0.00	46.73
011_D3	3778	56.10	0.00	0.00	43.90
011_D3	3895	0.00	25.20	74.80	0.00
011_D3	4024	54.88	0.00	0.00	45.12
011_D3	4177	79.84	20.16	0.00	0.00
011_D3	4213	13.37	0.00	86.63	0.00
011_D3	4219	0.00	32.44	67.56	0.00
011_D3	4228	0.00	51.56	0.00	48.44
011_D3	5275	0.00	48.00	52.00	0.00
011_D3	5842	0.00	73.08	26.92	0.00
011_D3	5929	0.00	26.41	73.59	0.00
011_D3	6061	0.00	70.11	29.89	0.00
011_D3	6124	0.00	22.22	77.78	0.00
011_D3	6250	21.60	0.00	0.00	78.40
011_D3	6259	0.00	48.15	51.85	0.00
011_D3	6343	0.00	23.57	76.43	0.00
011_D3	7513	32.09	0.00	0.00	67.91
011_D3	7546	69.56	0.00	0.00	30.44
011_D3	7588	0.00	20.63	79.37	0.00

011_D3	7648	0.00	37.25	62.75	0.00
011_D3	7816	0.00	79.43	20.57	0.00
011_D3	8014	0.00	39.88	60.12	0.00
011_D3	8059	34.70	0.00	0.00	65.30
011_D3	8419	0.00	77.09	22.91	0.00
011_D3	8692	0.00	69.98	30.02	0.00
011_D3	8716	78.84	0.00	0.00	21.16
011_DA	546	0.00	44.50	55.50	0.00
011_DA	1165	23.96	0.00	0.00	76.04
011_DA	2002	0.00	29.26	70.74	0.00
011_DA	2131	0.00	20.32	79.68	0.00
011_DA	2380	0.00	46.54	53.46	0.00
011_DA	2389	0.00	58.30	41.70	0.00
011_DA	2500	31.51	0.00	0.00	68.49
011_DA	2807	59.55	0.00	0.00	40.45
011_DA	3778	58.53	0.00	0.00	41.47
011_DA	4024	44.19	0.00	0.00	55.81
011_DA	4177	83.17	16.83	0.00	0.00
011_DA	4219	0.00	28.73	71.27	0.00
011_DA	4228	0.00	61.09	0.00	38.91
011_DA	5275	0.00	47.92	52.08	0.00
011_DA	5929	0.00	21.43	78.57	0.00
011_DA	6061	0.00	73.59	26.41	0.00
011_DA	6259	0.00	44.60	55.40	0.00
011_DA	6442	20.85	0.00	0.00	79.15
011_DA	7513	29.38	0.00	0.00	70.62
011_DA	7546	74.05	0.00	0.00	25.95
011_DA	7648	0.00	34.10	65.90	0.00
011_DA	8014	0.00	35.37	64.63	0.00
011_DA	8059	30.95	0.00	0.00	69.05
011_DA	8419	0.00	79.98	20.02	0.00
011_DA	8692	0.00	76.44	23.56	0.00
011_DA	9055	0.00	31.53	68.47	0.00
015_D0	1564	67.22	0.00	0.00	32.78
015_D0	2254	79.00	0.00	0.00	21.00
015_D0	3007	0.00	27.83	72.17	0.00
015_D0	3268	0.00	24.04	75.96	0.00
015_D0	4600	0.00	74.39	25.61	0.00
015_D0	5833	0.00	52.42	47.58	0.00
015_D0	7145	0.00	69.66	30.34	0.00
015_D0	7660	40.86	0.00	0.00	59.14
015_D0	8647	0.00	71.63	28.37	0.00
015_D0	9121	0.00	27.59	0.00	72.41
015_D3	1564	53.97	0.00	0.00	46.03
015_D3	4600	0.00	79.52	20.48	0.00
015_D3	7145	0.00	63.83	36.17	0.00
015_D3	7660	47.39	0.00	0.00	52.61
015_D3	8647	0.00	69.12	30.88	0.00

Table S4: Summary statistics of polymorphic sites in human pegivirus-1.

Sample	Genome assembly size (nt)	Total number of nucleotides				Number of polymorphic sites (% of the total number of polymorphic sites)						Normalised number of polymorphic sites (X/Y variant sites per 1000 sites of X+Y)					
		A	T	C	G	A/T	A/C	A/G	T/C	T/G	C/G	A/T	A/C	A/G	T/C	T/G	C/G
002_DA	8714	1569	1999	2370	2776	2 (2.47%)	0 (0%)	28 (34.57%)	50 (61.73%)	1 (1.23%)	0 (0%)	0.56	0.00	6.44	11.44	0.21	0.00
002_D0	9082	1626	2091	2468	2897	2 (2.38%)	0 (0%)	30 (35.71%)	51 (60.71%)	1 (1.19%)	0 (0%)	0.54	0.00	6.63	11.19	0.20	0.00
002_D3	9080	1631	2090	2470	2889	3 (3.57%)	0 (0%)	28 (33.33%)	52 (61.9%)	1 (1.19%)	0 (0%)	0.81	0.00	6.19	11.40	0.20	0.00
011_DA	9273	1672	2102	2535	2964	1 (3.85%)	0 (0%)	9 (34.62%)	15 (57.69%)	1 (3.85%)	0 (0%)	0.26	0.00	1.94	3.23	0.20	0.00
011_D0	9112	1646	2081	2473	2912	1 (2.33%)	0 (0%)	12 (27.91%)	29 (67.44%)	1 (2.33%)	0 (0%)	0.27	0.00	2.63	6.37	0.20	0.00
011_D3	9096	1648	2072	2472	2904	1 (2.7%)	2 (5.41%)	12 (32.43%)	21 (56.76%)	1 (2.7%)	0 (0%)	0.27	0.49	2.64	4.62	0.20	0.00
015_D0	9232	1667	2092	2535	2938	0 (0%)	0 (0%)	3 (30%)	6 (60%)	1 (10%)	0 (0%)	0.00	0.00	0.65	1.30	0.20	0.00
015_D3	9336	1685	2118	2565	2968	0 (0%)	0 (0%)	2 (40%)	3 (60%)	0 (0%)	0 (0%)	0.00	0.00	0.43	0.64	0.00	0.00

Supplementary Figures

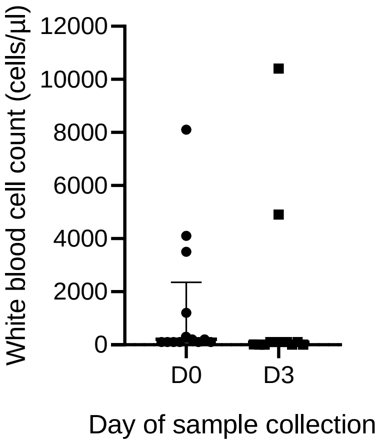

Figure S1: White blood cell counts on Day 0 (D0) and Day 3 (D3). D0: low quartile = 100 cells/µl; median = 200 cells/µl, upper-quartile = 2350 cells/µl. D3: low quartile = 0 cells/µl; median = 100 cells/µl, upper-quartile = 100 cells/µl.

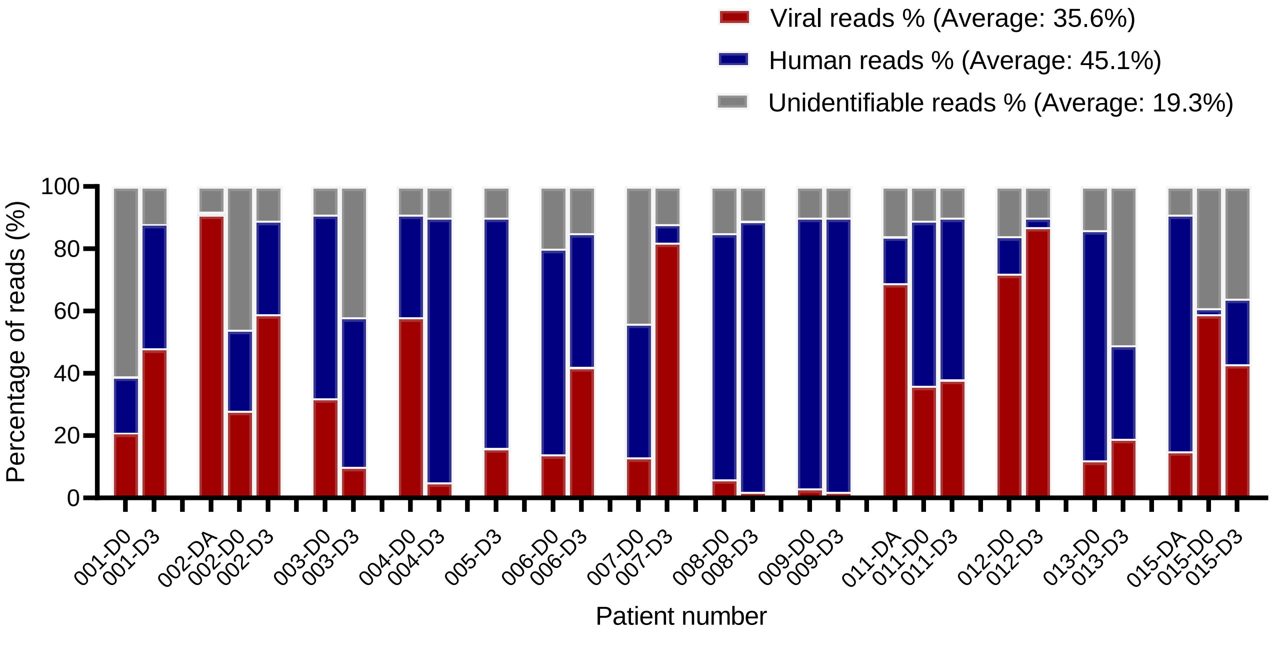

Figure S2: Frequencies of viral (red), human (blue), and unidentifiable reads (grey) as determined by Virus Identification Pipeline [1]. The actual numbers can be found in **Table S2**.

Supplementary Methods S1
Transplantation procedure

Graft-versus-host-disease (GVHD) prophylaxis

- Matched related and unrelated donor transplant

Two to 4 days before transplantation, patients received cyclosporin at a dose that was adjusted to achieve a plasma concentration of 250-350 ng/mL or tacrolimus at a dose that was adjusted to achieve a plasma concentration of 5-15 ng/mL. All received a short course of methotrexate.

- Haploidentical donor transplant

The GVHD prophylaxis consisted of cyclophosphamide 50 mg/kg/d on days SCT +3 (3rd day post Stem Cell Transplantation) and SCT +4, and on day SCT +5, tacrolimus or sirolimus was started (total administration time: 6 to 12 months), together with mycophenolate mofetil, 15 mg/kg orally twice daily for 60 days.

Supportive care

In our pediatric HSCT program, all patients were nursed in single rooms with a high-efficiency particulate air filter system. Antimicrobial prophylaxis consisted of oral itraconazole, penicillin V, and ciprofloxacin. Oral co-trimoxazole was started when the absolute neutrophil count was above 1.0×10^9 per liter. Antiviral prophylaxis consisted of daily acyclovir until the cessation of immunosuppressive agents.

Supplementary Methods S2

Preparation of the internal control

Newcastle disease virus (NDV) was isolated from a chicken cloacal swab sample collected at a slaughterhouse, Mukdahan province, Thailand in June 2017. The virus was isolated by inoculating specific-pathogen-free (SPF) ECE and it was confirmed by plate HA inhibition (HI) test. Then, the isolated virus was propagated using MDCK (Madin-Darby canine kidney) cell line (ATCC NBL -2). Viral RNA from culture supernatant was extracted using RNeasy mini kit (Qiagen, Hilden, Germany) and used one-step PCR kit (Qiagen, Hilden, Germany) for viral quantification by real-time RT-PCR. The PCR conditions are given in Supplementary Methods 3. Next, the culture supernatant was divided into portions and stored in sterile screw-capped vials at -80°C.

Supplementary Methods S3

qRT-PCR protocols

The NDV primers used for PCR amplification were 5'-GGAGGATGTTGGCAGCATT-3' and 5'-GTCAACATATACACCTCATC-3', which resulted in a product of 318 bp [2]. Initial denaturation was done at 95°C for 15 minutes, followed by 35 cycles of denaturation at 94°C for 30 seconds, annealing at 58°C for 30 seconds, and extension at 72°C for 30 seconds, and then melting curve analysis from 65°C to 95°C.

The HPgV-1 primers used for PCR amplification were 5'-GGTCGTAAATCCCGGTCACC-3' and 5'-CCCACTGGTCCTTGTCAACT-3' which targeted the 5' untranslated region (UTR) [3]. PCR cycle included 30 minutes of reverse transcription at 50°C, 10 minutes of initial PCR activation at 95°C, followed by 50 cycles of denaturation at 95°C

for 20 seconds, annealing at 57°C for 20 seconds, and extension at 72°C for 20 seconds and then melting curve analysis from 65°C to 95°C.

References

1. Li, Y.; Wang, H.; Nie, K.; Zhang, C.; Zhang, Y.; Wang, J.; Niu, P.; Ma, X. VIP: an integrated pipeline for metagenomics of virus identification and discovery. *Scientific reports* **2016**, *6*, 1-10.

2. Stäuber, N.; Brechtbühl, K.; Bruckner, L.; Hofmann, M.A. Detection of Newcastle disease virus in poultry vaccines using the polymerase chain reaction and direct sequencing of amplified cDNA. *Vaccine* **1995**, *13*, 360-364.

3. Shahzamani, K.; Jahanbakhsh, S.; Lashgarian, H. Qualitative detection of GB Virus C and Hepatitis C Virus co-infection in cirrhotic patients using a SYBR green multiplex RT-PCR technique. *Tropical Biomedicine* **2017**, *34*, 822-830.
